# 

**Published:** 1908-02

**Authors:** 


PUBLISHED MONTHLY. | SUBSCRIPTION $1.00

### ATLANTA JOURNAL-RECORD OF MEDICINE

LIBRARY
FEB. 24. 1908

Successor to { Atlanta Medical and Surgical Journal, Established 1855. and Southern Medical Record, Established 1870. } 52d Year

Vol. IX. ATLANTA, GA., FEBRUARY, 1908. No. 11

